# Origin of the neonatal gut microbiota and probiotic intervention: a randomized controlled trial

**DOI:** 10.3389/fnut.2024.1389417

**Published:** 2024-04-30

**Authors:** Zhe Li, Yiwen Zhang, Xiaozhi Tan, Tye Kian Deng, Qian Gao, Xiaomin Xiao, Chengfang Xu

**Affiliations:** ^1^Department of Obstetrics, The Third Affiliated Hospital of Sun Yat-sen University, Guangzhou, Guangdong, China; ^2^Department of Obstetrics, The Fifth Affiliated Hospital of Guangzhou Medical University, Guangzhou, Guangdong, China; ^3^Department of Obstetrics and Gynecology, First Affiliated Hospital of Jinan University, Guangzhou, Guangdong, China

**Keywords:** gut microbiota, vaginal microbiota, placenta microbiota, neonatal gut microbiota, probiotic

## Abstract

**Objective:**

This study aims to evaluate the origin of the neonatal gut microbiota on the 14th day and probiotic intervention in the third trimester.

**Methods:**

Samples were obtained from a total of 30 pregnant individuals and their offspring, divided into a control group with no intervention and a probiotic group with live combined *Bifidobacterium* and *Lactobacillus* tablets, analyzing by 16S rRNA amplicon sequencing of the V4 region to evaluate the composition of them. Non-metric multidimensional scaling and SourceTracker were used to evaluate the origin of neonatal gut microbiota.

**Results:**

We found that the microbiota in the neonatal gut at different times correlated with that in the maternal microbiota. The placenta had more influence on meconium microbiota. Maternal gut had more influence on neonatal gut microbiota on the 3rd day and 14th day. We also found that the maternal gut, vaginal, and placenta microbiota at full term in the probiotic group did not have a significantly different abundance of *Bifidobacterium*, *Lactobacillus,* or *Streptococcus*. However, some other bacteria changed in the maternal gut and their neonatal gut in the probiotic group.

## Introduction

There are billions of microbial cells in the human body, which are vital to human health (1, 2). These bacteria are found at the highest density in the intestinal tract and are called the gut microbiota (1). The gut microbiota tends to be stable from the neonatal period to adulthood (1, 3). The neonatal gut microbiota is complicated and influenced by the placenta, umbilical cord, and amniotic fluid, so the foundation of the neonatal gut microbiota may begin from the neonate during the gestational period (4, 5). The gut microbiota of pregnant women may induce premature delivery (6). The gut microbiota of neonates can lead to allergic asthma in childhood by influencing the function of immune cells (7). This relationship was also found in children with infantile intestinal disease or other diseases (7, 8).

Recent research has shown that enterococcus species that are orally administered to pregnant mice can be detected via genetic markers in the meconium of offspring delivered by cesarean section (4). Another study first described the molecular mechanism through which the maternal gut microbiome can regulate nervous system development in mice (9). These findings showed the possibility of manual intervention of offspring’s gut microbiota. *Bifidobacterium* species are vital to human health and are used in combination with probiotics (10). Some research has shown that *Bifidobacterium* can be vertically transmitted from mother to infant (11). A published RCT showed that oral probiotics administered during the gestational period can reduce the incidence of neonatal allergic disease, although the underlying mechanism is still unclear (12). Therefore, determining the origin of the neonatal gut microbiota and evaluating the potential application of probiotic intervention are necessary.

## Materials and methods

### Study design and participants

A total of 31 pregnant women receiving antenatal care at the 1st Affiliated Hospital of Jinan University were recruited in this study and randomly assigned to the probiotic group (*n* = 15) and the control group (*n* = 16) (NCT06241222). Informed consent was obtained from pregnant women (at least 32 gestational weeks) who met the inclusion criteria. Newborns were followed until 14 days after natural delivery, and all newborns in our study were breastfed. During follow-up, one pregnant woman in the probiotic group was excluded due to loss of follow-up. Pregnant inclusive criteria were as follows: 1. Chinese woman who is pregnant with a single fetus; and 2. First pregnancy and term delivery. Newborns’ inclusive criteria were as follows: 1. Normal weight (>2,500 g, <4,000 g); 2. Term infant (>37 weeks, <42 weeks); and 3. Natural birth. Pregnant exclusive criteria were as follows: 1. Gastrointestinal disease or family history; 2. Vaginitis before pregnancy; 3. Antibiotic usage during pregnancy; 4. Hypertension, diabetes mellitus, hyperthyroidism, hypothyroidism, autoimmune disease, or other endocrine and metabolic disease; 5. Gestational hypertensive disease, gestational diabetes mellitus, or other gestational disease; 6. Transfusion history, organ transplantation history or immunotherapy history; and 7. Other probiotics or prebiotics usage during pregnancy. Newborn’s exclusive criteria were as follows: 1. Abnormal weight (>4,000 g, <2,500 g); 2. With congenital diseases; and 3. Intrapartum fetal complication.

### Probiotic management

In our study, 14 pregnant women were randomly assigned to the probiotic group, and the rest were assigned to the control group. After enrollment, pregnant women in the probiotic group received a combination of living *Bifidobacterium longum* (1 ∗ 10^8^ CFU, no less than 5 ∗ 10^6^ CFU), *Lactobacillus delbrueckii bulgaricus* (1 ∗ 10^7^ CFU, no less than 5 ∗ 10^5^ CFU), and *Streptococcus thermophilus* (1 ∗ 10^7^ CFU, no less than 5 ∗ 10^5^ CFU) tablets produced by Inner Mongolia Shuangqi Pharmaceutical Co., Ltd. PRC. Pregnant individuals in the probiotic group were administered two tablets twice a day (2 g/d) until natural delivery, and those in the control group took no pills.

### Fecal, vaginal secretion, placental, and meconium collection

#### Fecal collection

Feces from pregnant patients were collected twice—between weeks 32 and 34 and before natural labor. Feces collected at weeks 32–34 were collected internally, thereby avoiding contamination with foreign material. The feces were stored in a domestic refrigerator and transferred to the laboratory freezer at −80°C for 24 h. Feces collected before labor were obtained in the hospital and transferred to the laboratory freezer within 30 min of collection (13, 14).

#### Vaginal secretion collection

Vaginal secretions were collected twice between weeks 32 and 34 and before natural labor. The pregnant woman was asked to not engage in sexual behavior, clean the vulva, clean the vagina, or use vaginal medicine within 48 h before sample collection. Vaginal secretions before natural labor were collected before membrane rupture occurred. The samples were transferred to the laboratory freezer within 30 min of collection, thereby avoiding contamination by foreign material.

#### Placenta collection

Placenta samples ranging from the umbilical cord to 3 cm were collected by stripping the amniotic membrane after a natural birth. The blood was rinsed with sterile saline. Four to six pieces of placenta were sampled from the fetal surface. Each piece had a volume of approximately 1 cm^3^. The placenta sample was transferred to the laboratory freezer within 30 min of collection.

#### Meconium collection

Meconium was collected three times on the 1st day, 3rd day, and 14th day after a natural delivery. On the 1st day and 3rd day, meconium was collected internally, thereby avoiding contamination with foreign material in the hospital, and was transferred to the laboratory freezer within 30 min of collection. On the 14th day, meconium was collected, stored in a domestic refrigerator, and then transferred to a laboratory freezer within 24 h of collection.

### Experimental procedures

#### DNA extraction and sample quality control

For the soil, feces, and intestinal content samples, DNA was extracted by using a Magnetic Soil and Stool DNA Kit (TianGen, China, Catalog #: DP712). For other types of samples, DNA was extracted by using the CTAB extraction method. Please refer to the QC Report for the sample quality control methods.

### Amplicon generation

16S rRNA/18SrRNA/ITS genes of distinct regions (16SV4/16SV3/16SV3- V4/16SV4- V5, 18SV4/18SV9, ITS1/ITS2, and ArcV4) were amplified using a specific primer (e.g., 16SV4: 515F- 806R, 18SV4: 528F-706R, and 18SV9: 1380F- 1510R) with a barcode. All PCRs were conducted with 15 μL of Phusion® High-Fidelity PCR Master Mix (New England Biolabs), 0.2 μM forward and reverse primers, and approximately 10 ng of template DNA. Thermal cycling consisted of initial denaturation at 98°C for 1 min, followed by 30 cycles of denaturation at 98°C for 10 s, annealing at 50°C for 30 s, elongation at 72°C for 30 s, and extension at 72°C for 5 min.

### PCR product quantification and qualification

The same volume of 1X loading buffer (containing SYB green) was mixed with the PCR products, and electrophoresis was performed on a 2% agarose gel for detection. The PCR products were mixed in ratios with equal densities. The mixture of PCR products was subsequently purified with a Universal DNA Purification Kit (TianGen, China, Catalog #: DP214).

### Library preparation and sequencing

Sequencing libraries were generated using the NEBNext® Ultra™ II FS DNA PCR-free Library Prep Kit (New England Biolabs, USA, Catalog#: E7430L) according to the manufacturer’s recommendations, and indexes were added. The library was checked with a Qubit fluorometer and real-time PCR for quantification, and a bioanalyzer was used to assess the size distribution. The quantified libraries were pooled and sequenced on an Illumina platform based on the effective library concentration and data amount.

### Data analysis

Data analysis is performed using the single-end reads assembly and quality control.

### Data split

Single-end reads were assigned to samples based on their unique barcode and truncated by cutting off the barcode and primer sequence.

### Data filtration

Quality filtering on the raw reads was performed under specific filtering conditions to obtain the high-quality clean reads according to the Cutadapt (Martin M., 2011) (V1.9.1^1^), quality-controlled process.

### Chimera removal

The reads were compared with the reference database (Gold database^2^) using the UCHIME algorithm (UCHIME Algorithm^3^) to detect chimera sequences, and then the chimera sequences were removed. Then, the effective tags were finally obtained.

### Operational taxonomic units (OTUs) cluster and species annotation

#### OTU production

Sequence analysis was performed by Uparse software (Uparse v7.0.1001^4^). Sequences with ≥97% similarity were assigned to the same OTUs. The representative sequence for each OTU was screened for further annotation.

### Species annotation

For each representative sequence, the Silva Database^5^ was used based on the RDP classifier (Version 2.2^6^) algorithm to annotate taxonomic information.

### Phylogenetic relationship construction

To study the phylogenetic relationship of different OTUs and the difference of the dominant species in different samples (groups), multiple sequence alignments were conducted using the MUSCLE software (Version 3.8.31^7^).

### Data normalization

OTUs abundance information was normalized using a standard sequence number corresponding to the sample with the least sequences. Subsequent analyses of alpha diversity and beta diversity were all performed based on this output normalized data.

### Alpha diversity

Alpha diversity is applied in analyzing the complexity of species diversity for a sample through Shannon, calculated with QIIME (Version 1.7.0) and displayed with R software (Version 2.15.3) to identify community richness. Shannon—the Shannon index.^8^

### Beta-diversity

To evaluate the complexity of the community composition and compare the differences between samples (groups), beta diversity was calculated based on weighted and unweighted unifrac distances in QIIME. Non-metric multidimensional scaling (NMDS) was also implemented for data dimension reduction. NMDS uses the distance matrix, but it emphasizes the numerical rank instead. NMDS analysis was implemented through R software with the ade4 package and ggplot2 package.

### Source tracker

To estimate the proportion of a microbiota community that comes from a set of source environments, SourceTracker (v1.0), a Bayesian approach, was used. Neonatal gut microbiota was designed as sink, and their maternal gut, vaginal, and placenta microbiota were designed as sources (15).

### Co-occurrence network analysis

Spearman’s rank correlation coefficient was calculated using the R package of “ccrepe” between genera based on the relative abundance profile of genera. Networks were then constructed by using the method implemented in Cytoscape (v3.6) (16).

### Statistical analysis

The significant difference between the sample communities was determined through linear discriminant analysis of effect size (LEfSe). A *p* < 0.05 was considered statistically significant. Spearman’s rank correlation between maternal microbiota and neonatal microbiota was analyzed using the stats package and ggplot2 package in R software with a significance level of *p* < 0.05.

## Results

### The origin of the neonatal gut microbiota

**Figure 1:**
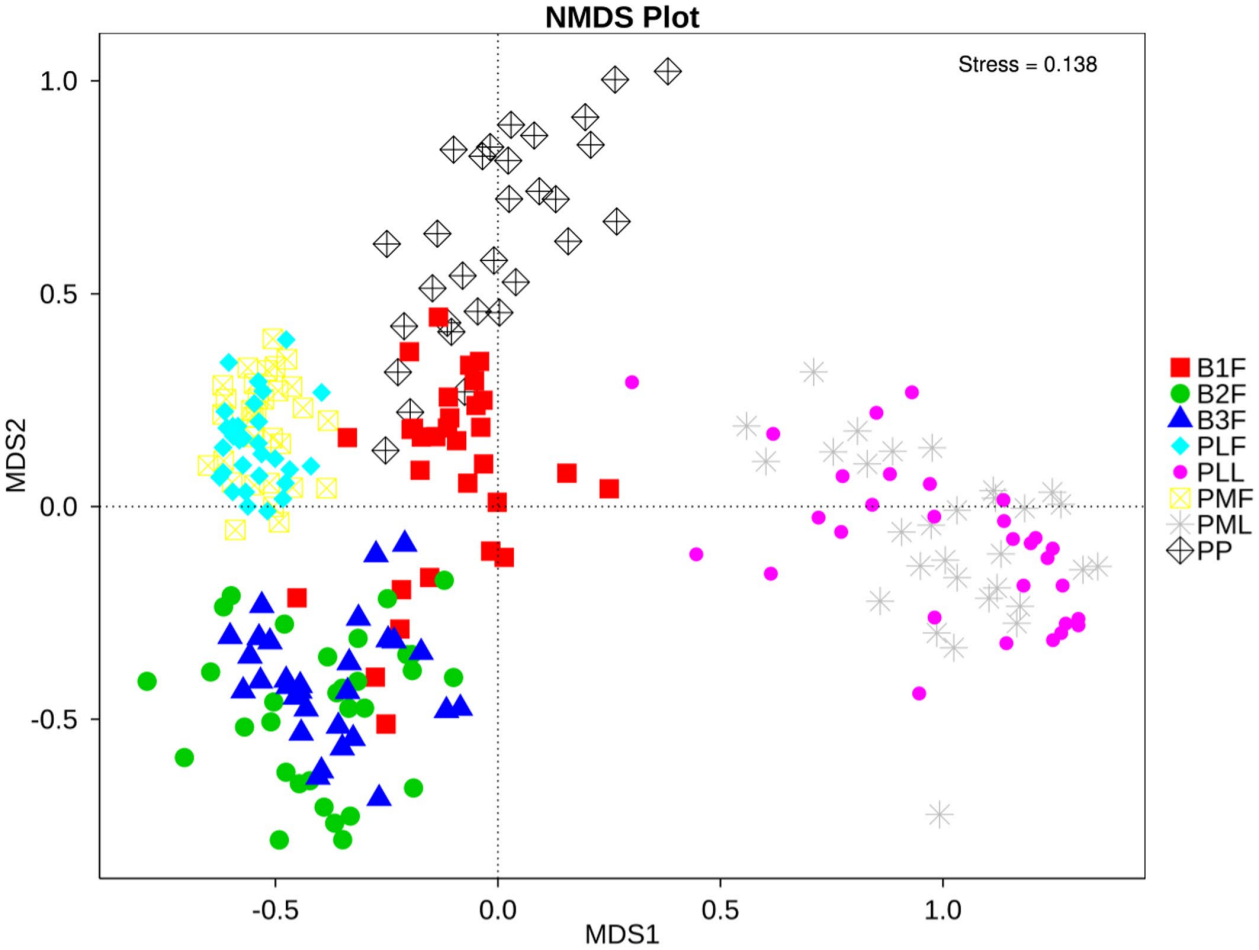
NMDS plot of each sample. PMF, gut microbiota at gestational 32–34 weeks women; PML, vaginal microbiota at gestational 32–34 weeks women; PLF, gut microbiota of gestational full-term women; PLL, vaginal microbiota of gestational full-term women; PP, placenta microbiota; B1F, meconium microbiota; B2F, the gut microbiota of newborns on the 3rd day; B3F, the gut microbiota of newborns on the 14th day. The distance of each point presented the degrees of difference of each sample’s microbiota.

Non-metric multidimensional scaling (NMDS) was used to analyze the diversity of microbiota in each group. Neonatal gut microbiota was more similar to the placenta and maternal gut microbiota (Figure 1).

**Figure 2:**
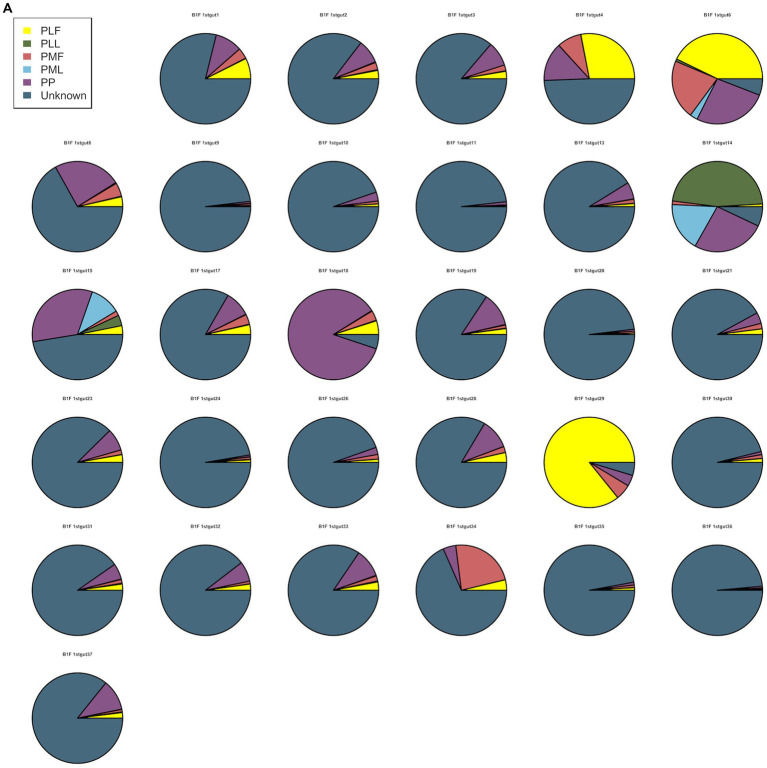

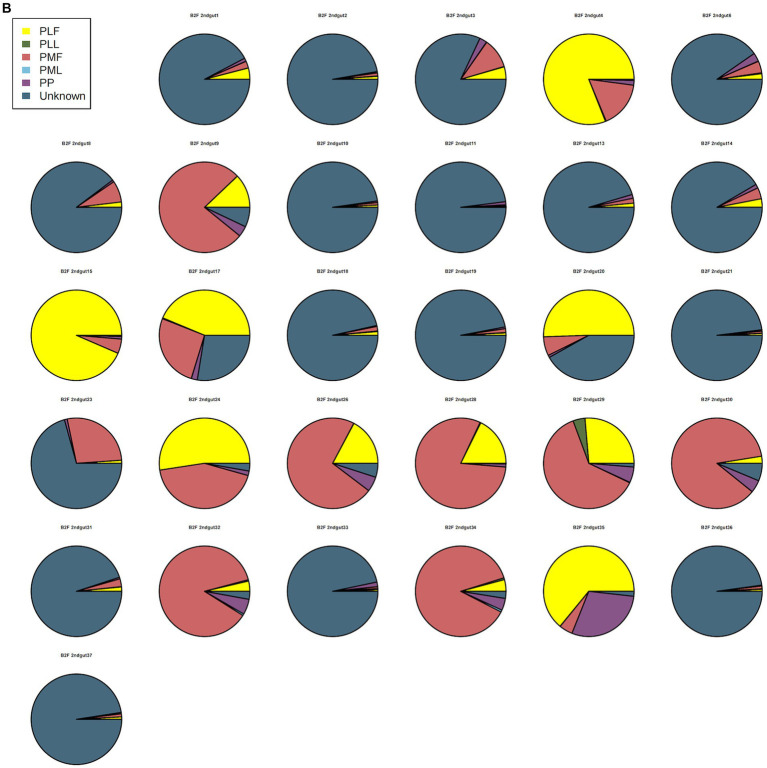

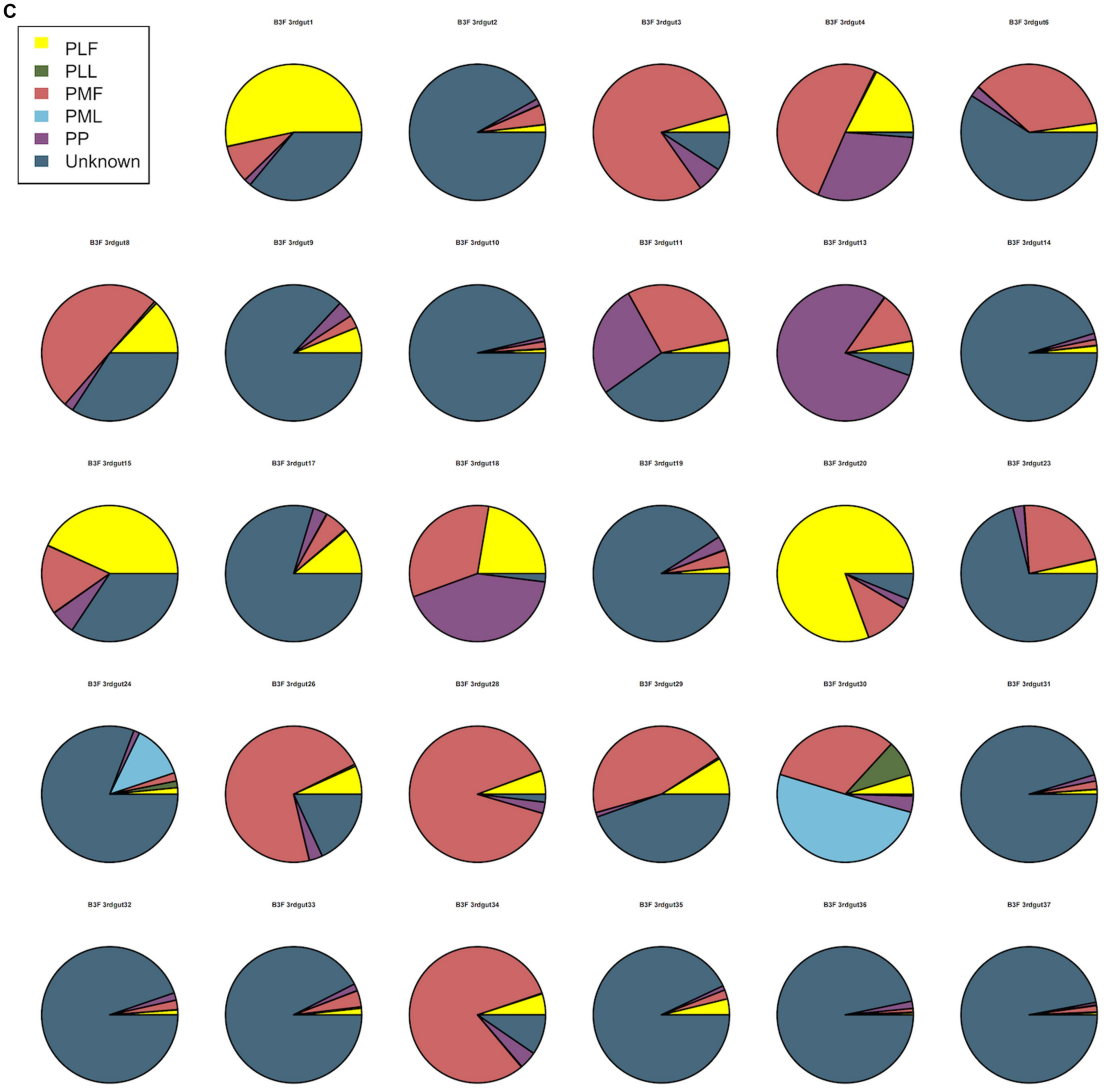
**(A)** SourceTracker of gut microbiota in B1F. **(B)** SourceTracker of gut microbiota in B2F. **(C)** SourceTracker of gut microbiota in B3F. PMF, gut microbiota at gestational 32–34 weeks women; PML, vaginal microbiota at gestational 32–34 weeks women; PLF, gut microbiota of gestational full-term women; PLL, vaginal microbiota of gestational full-term women; PP, placenta microbiota; B1F, meconium microbiota; B2F, the gut microbiota of newborns on the 3rd day; B3F, the gut microbiota of newborns on the 14th day.

Neonatal gut microbiota proportions were estimated using SourceTracker and samples from the maternal gut, vagina, and placenta. In general, meconium microbiota communities were composed mainly of bacteria from the placenta and an unknown source (Figure 2A). As to neonatal gut microbiota on the 3rd day, it tended to be composed mainly of bacteria from maternal gut microbiota (including 32–34 weeks and full term) and an unknown source (Figure 2B). On the 14th day, neonatal gut microbiota were composed mainly of bacteria from maternal gut microbiota (especially in 32–34 weeks) and an unknown source (Figure 2C).

### The relationship between bacteria from pregnant women and their offspring

**Figure 3:**
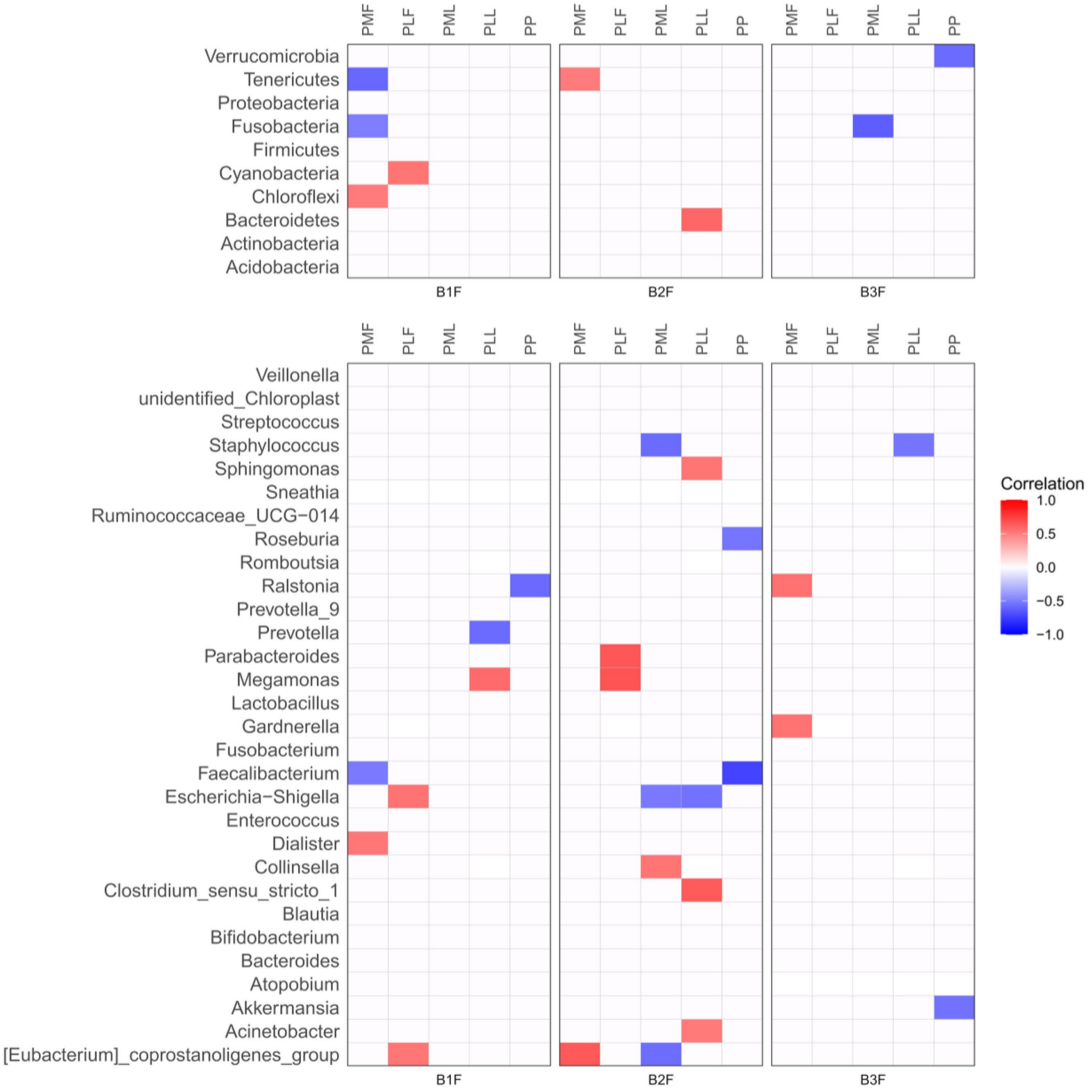
Spearman correlation heatmap of microbiota in pregnancies and their offspring. PMF, gut microbiota at gestational 32–34 weeks women; PML, vaginal microbiota at gestational 32–34 weeks women; PLF, gut microbiota of gestational full-term women; PLL, vaginal microbiota of gestational full-term women; PP, placenta microbiota; B1F, meconium microbiota; B2F, the gut microbiota of newborns on the 3rd day; B3F, the gut microbiota of newborns on the 14th day. Red color represented a positive correlation and blue color represented a negative correlation.

Spearman correlation heatmap was used to show the microbiota correlation between pregnant women and their offspring in phylum (Top 10) and genus level (Top 30) (Figure 3).

At the phylum level, *Chloroflexi* in meconium microbiota (B1F) had a positive correlation and *Fusobacteria* and *Tenerivutes* in B1F had a negative correlation with those in gut microbiota at gestational 32–34 weeks women (PMF). *Cyanobacteria* in B1F had a positive correlation with those in the gut microbiota of gestational full-term women (PLF). *Tenericutes* in the gut microbiota of newborns on the 3rd day (B2F) had a positive correlation with those in PMF. *Bacteroidetes* in B2F had a positive correlation with those in the vaginal microbiota of gestational full-term women (PLL). *Fusobacteria* in the gut microbiota of newborns on the 14th day (B3F) had a negative correlation with those in vaginal microbiota at gestational 32–34 weeks women (PML). *Verrucomicrobia* in B3F had a negative correlation with those in placenta microbiota (PP).

At the genus level, *Dialister* in B1F had a positive correlation and *Faecalibacterium* in B1F had a negative correlation with those in PMF. *Escherichia-Shigella* and *[Eubacterium]_coprostanoligenes_group* in B1F had a positive correlation with those in PLF. *Meganonas* in B1F had a positive correlation and *Prevotella* in B1F had a negative correlation with those in PLL. *Ralstonia* in B1F had a negative correlation with those in PP. *[Eubacterium]_coprostanoligenes_group* in B2F had a positive correlation with those in PMF. *Parabacteroides and Megamonas* in B2F had a positive correlation with those in PLF. *Collinsella* in B2F had a positive correlation, *Staphylococcus*, *Escherichia-Shigella,* and *[Eubacterium]_coprostanoligenes_group* in B2F had a negative correlation with those in PML. *Sphingomonas*, *Clostridium_sensu_stricto_1,* and *Acinetobacter in* B2F had a positive correlation and *Escherichia-Shigella* in B2F had a negative correlation with those in PLL. *Roseburia* and *Faecalibacterium* in B2F had a negative correlation with those in PP. *Ralstonia* and *Gardnerella* in B3F had a positive correlation with those in PMF. *Staphylococcus* in B3F had a negative correlation with those in PLL. *Akkermansia* in B3F had a negative correlation with those in PP.

### Network diagram of the correlation of differential microbiota

**Figure 4:**
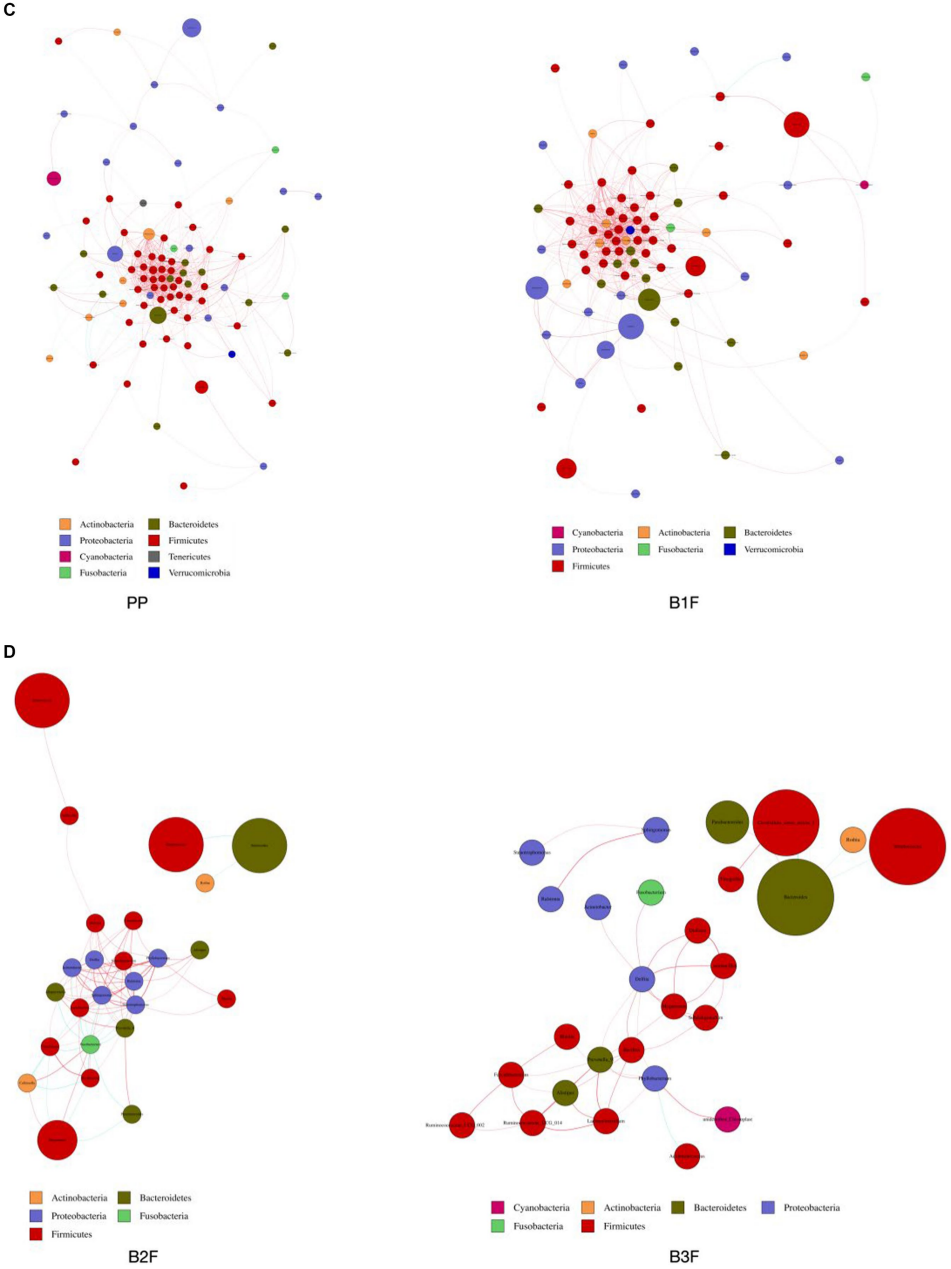
**(A)** Co-occurrence network of maternal gut microbiota. **(B)** Co-occurrence network of maternal vaginal microbiota. **(C)** Co-occurrence network of placenta microbiota and meconium microbiota. **(D)** Co-occurrence network of neonatal gut microbiota on the 3rd day and 14th day. PMF, gut microbiota at gestational 32–34 weeks women; PML, vaginal microbiota at gestational 32–34 weeks women; PLF, gut microbiota of gestational full-term women; PLL, vaginal microbiota of gestational full-term women; PP, placenta microbiota; B1F, meconium microbiota; B2F, the gut microbiota of newborns on the 3rd day; B3F, the gut microbiota of newborns on the 14th day. Co-occurrence network analysis was based on core genus (average relative abundance > 0.005%). Each node represents each species, node color represents phylum, and node size represents relative OTUs they had. The connecting line represents the existence of a significant correlation between two nodes, Spearman correlation coefficient value below 0 (negative correlation) indicates the green line, Spearman correlation coefficient value greater than 0 (positive correlation) indicates the red line. The thicker the line, the greater the Spearman correlation coefficient between the two nodes.

We constructed the co-occurrence network of the core genera in maternal microbiota and neonatal microbiota. From this diagram, we see that bacteria have a complicated correlation with other bacteria in the maternal gut, vagina, placenta, and neonatal gut in different stages. The correlation of bacteria in the placenta and meconium was the most complicated. In 32–34 weeks and full term, *Bifidobacterium*, *Lactobacillus,* and *Streptococcus* had complicated relationships with other bacteria. Neonatal gut microbiota’s correlation became uncomplicated gradually from 0 days to 14 days (Figures 4A–D).

### Microbiota difference between the control group and the probiotic group

Clinical characteristics are recorded in Table 1.

**Table 1:** Clinical characteristics of pregnancies in different groups.

	Control group (Mean ± SD)	Probiotic group (Mean ± SD)	*P-*value
Maternal age	27.73 ± 4.50	27.20 ± 3.91	0.885
Maternal weight (kg)	65.54 ± 9.03	65.34 ± 9.28	0.561
Maternal height (cm)	162.53 ± 8.31	158.55 ± 3.82	0.060
Maternal BMI (kg/m^2^)	24.75 ± 2.29	25.94 ± 3.18	0.092
Neonatal weight (kg)	3.45 ± 0.71	3.34 ± 0.53	0.839
Neonatal height (cm)	49.73 ± 0.67	50.10 ± 1.66	0.938
Neonatal head circumference (cm)	35.60 ± 7.39	33.90 ± 0.99	0.179

*p < 0.05.

### Alpha diversity difference

**Figure 5:**
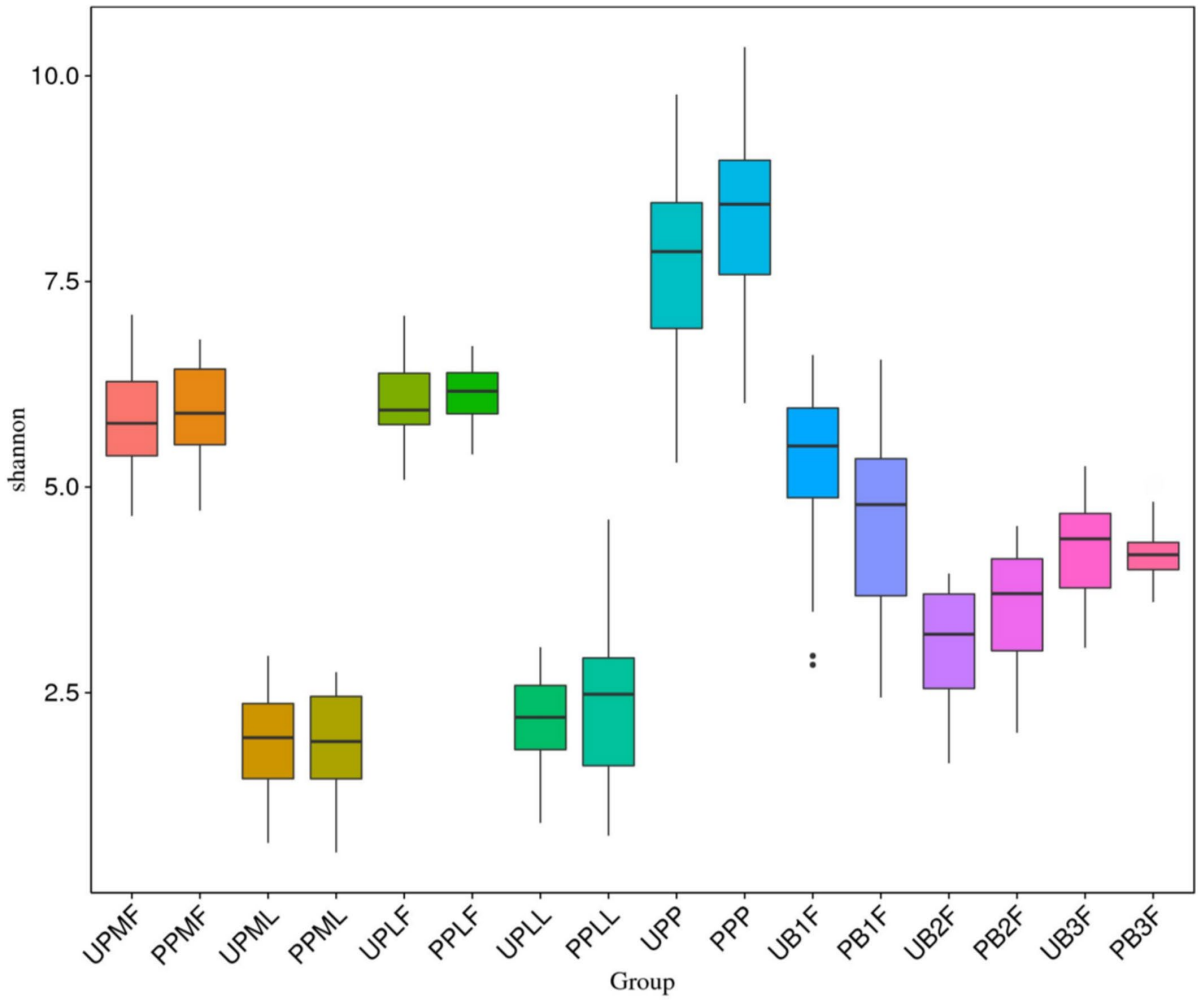
Comparison of alpha diversity (as assessed by the Shannon index) of each group. UPMF, gut microbiota at gestational 32–34 weeks women in control group; PPMF, gut microbiota at gestational 32–34 weeks women in probiotic group; UPML, vaginal microbiota at gestational 32–34 weeks in control group; PPML, vaginal microbiota at gestational 32–34 weeks in probiotic group; UPLF, gut microbiota of gestational full-term women in control group; PPLF, gut microbiota of gestational full-term women in probiotic group; UPLL, vaginal microbiota of gestational full-term women in control group; PPLL, vaginal microbiota of gestational full-term women in probiotic group; UB1F, meconium microbiota in control group; PB1F, meconium microbiota in probiotic group; UB2F, the gut microbiota of newborns on the 3rd day in control group; PB2F, the gut microbiota of newborns on the 3rd day in probiotic group; UB3F, the gut microbiota of newborns on the 14th day in control group; PB3F, the gut microbiota of newborns on the 14th day in probiotic group; UPP, placenta microbiota in control group; PPP, placenta microbiota in probiotic group.

The Shannon index was used to evaluate the species diversity index of microbiota. After probiotic treatment, the alpha diversity of meconium microbiota in the probiotic group was significantly lower than those of meconium microbiota in the control group (*p* < 0.05) (Figure 5).

**Figure 6:**
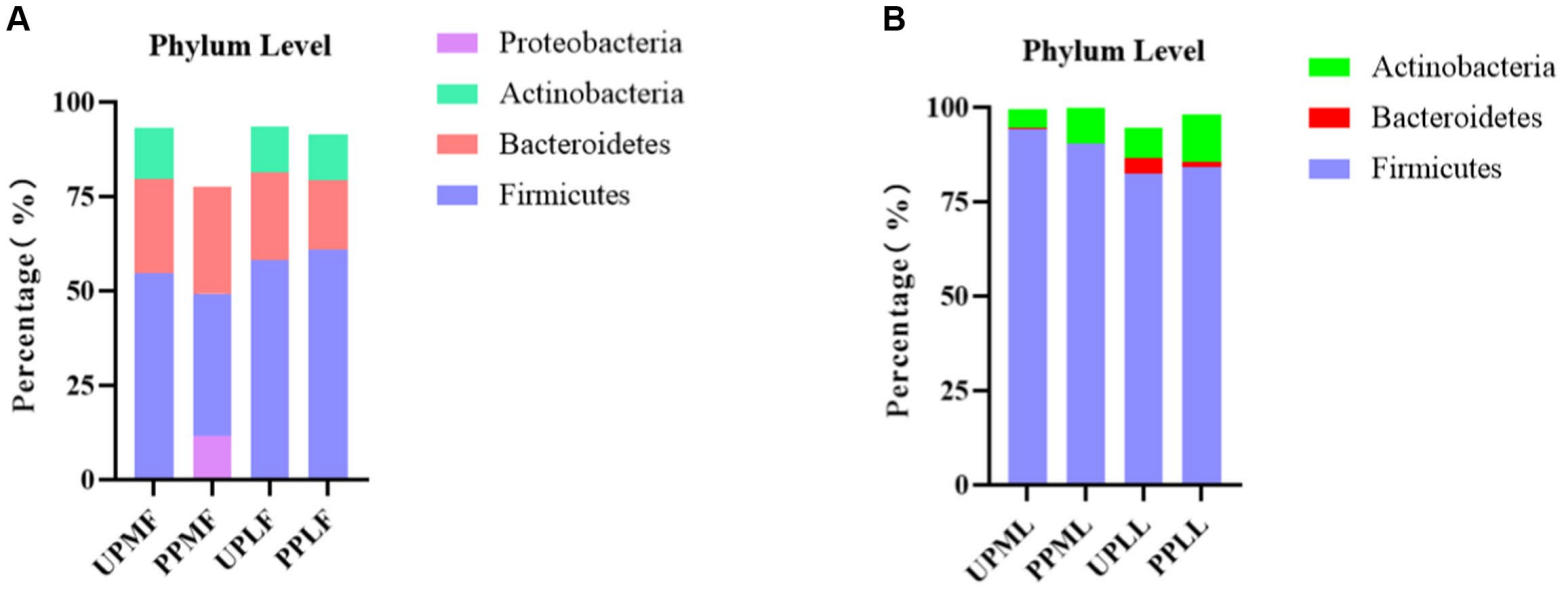
**(A,B)** Relative OTUs of the gut and vaginal microbiotas at the phylum level.

At the phylum level, *Firmicutes* (54.7%), *Bacteroidetes* (25.1%), and *Actinobacteria* (13.6%) were the three most common components of the pregnant gut microbiota at 32–34 weeks in the control group (gut microbiota at gestational 32–34 weeks women in the control group (UPMF)). *Firmicutes* (49.3%), *Bacteroidetes* (28.3%), and *Proteobacteria* (11.7%) were the three most common components of the gut microbiota at 32–34 weeks in the probiotic group before probiotics were administered (gut microbiota at gestational 32–34 weeks women in the probiotic group (PPMF)). The difference between the two groups was not significant (*p* > 0.05). *Firmicutes*, *Bacteroidetes,* and *Actinobacteria* were the three most abundant components of the pregnant gut microbiota in both the control group (gut microbiota of gestational full-term women in the control group (UPLF)) and the probiotic group after receiving the probiotic (gut microbiota of gestational full-term women in the probiotic group (PPLF)) at full term. The difference between the two groups was not significant (*p* > 0.05) (Table 2; Figure 6A).

**Table 2:** Relative OTUs of the gut and vaginal microbiotas at the phylum level (%).

	*Firmicutes*	*Bacteroidetes*	*Actinobacteria*	*Proteobacteria*
UPMF	54.7	25.1	13.6	
PPMF	49.3	28.3		11.7
UPLF	58.0	23.4	12.2	
PPLF	61.0	18.4	12.2	
UPML	94.2	0.4	4.9	
PPML	90.4	0.1	9.3	
UPLL	82.5	4.0	8.2	
PPLL	84.2	1.4	12.7	

UPMF, gut microbiota at gestational 32–34 weeks women in control group; PPMF, gut microbiota at gestational 32–34 weeks women in probiotic group; UPLF, gut microbiota of gestational full-term women in control group; PPLF, gut microbiota of gestational full-term women in probiotic group; UPML, vaginal microbiota at gestational 32–34 weeks in control group; PPML, vaginal microbiota at gestational 32–34 weeks in probiotic group; UPLL, vaginal microbiota of gestational full-term women in control group; PPLL, vaginal microbiota of gestational full-term women in probiotic group.

At the phylum level, *Firmicutes* (94.2%), *Actinobacteria* (4.9%), and *Bacteroidetes* (0.4%) were the three most abundant groups in the vaginal microbiota at 32–34 weeks in both the control group (vaginal microbiota at gestational 32–34 weeks in the control group (UPML)) and the probiotic group before probiotic taken (vaginal microbiota at gestational 32–34 weeks in the probiotic group (PPML)). *Firmicutes* (82.5%), *Actinobacteria* (8.2%), and *Bacteroidetes* (4.0%) were the three most abundant components of the vaginal microbiota in the control group (vaginal microbiota of gestational full-term women in the control group (UPLL)) at full term. *Firmicutes* (84.2%), *Actinobacteria* (12.7%), and *Teneria* (1.4%) were the three most abundant components of the vaginal microbiota in the probiotic group after probiotics were administered (vaginal microbiota of gestational full-term women in the probiotic group (PPLL)) at full term. The difference between the two groups was not significant (*p* > 0.05) (Table 2; Figure 6B).

### Variation in the gut and vaginal microbiota at the genus level in probiotic-treated pregnant patients

**Figure 7:**
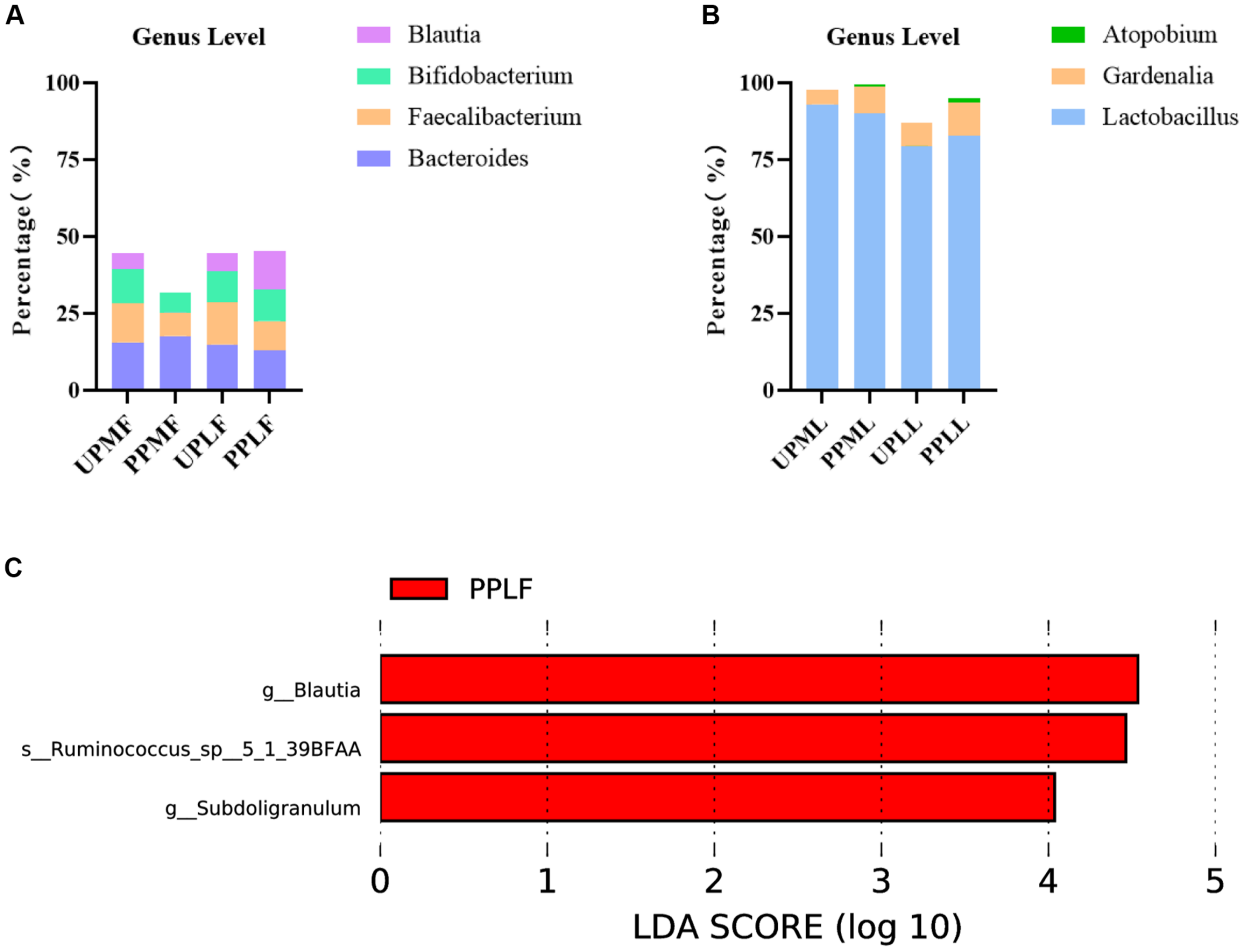
**(A,B)** Relative OTUs of the gut and vaginal microbiota at the genus level. **(C)** LDA analysis results for the gut microbiota at full term in the control group and probiotic group. UPMF, gut microbiota at gestational 32–34 weeks women in control group; PPMF, gut microbiota at gestational 32–34 weeks women in probiotic group; UPLF, gut microbiota of gestational full-term women in control group; PPLF, gut microbiota of gestational full-term women in probiotic group; UPML, vaginal microbiota at gestational 32–34 weeks in control group; PPML, vaginal microbiota at gestational 32–34 weeks in probiotic group; UPLL, vaginal microbiota of gestational full-term women in control group; PPLL, vaginal microbiota of gestational full-term women in probiotic group. p_: Phylum, c_: Class, o_: Order, f_: Family, g_: Genus. The length of the bars represents the magnitude of the impact of differential species (i.e., LDA score).

At the genus level, *Bacteroides* (15.6%), *Faecalibacterium* (12.7%), and *Bifidobacterium* (11.1%) were the three most abundant components of the gut microbiota in pregnant patients at 32–34 weeks in the control group (UPMF). *Bacteroides* (17.7%), *Escherichia* (8.2%), and *Faecalibacterium* (7.7%) were the three most abundant components of the gut microbiota in pregnant patients at 32–34 weeks in the probiotic group before probiotics were administered (PPMF). The difference between the two groups was not significant (*p* > 0.05). *Bacteroides* (14.9%), *Faecalibacterium* (13.8%), and *Bifidobacterium* (10.0%) were the three most common components of the gut microbiota at full term in the control group (UPLF). Among the pregnant patients, *Bacteroides* (13.0%), *Blautia* (12.3%), and *Bifidobacterium* (10.4%) were the three most common components of the gut microbiota at full term in the probiotic group after receiving probiotics (PPLF) (Table 3; Figure 7A).

**Table 3:** Relative OTUs of the gut and vaginal microbiota at the genus level (%).

	*Bacteroides*	*Faecalibacterium*	*Bifidobacterium*	*Blautia*	*Lactobacillus*	*Gardenalia*	*Atopobium*
UPMF	15.6	12.7	11.1	5.2			
PPMF	17.7	7.7	6.3				
UPLF	14.9	13.8	10.0	5.9			
PPLF	13.0	9.5	10.4	12.3			
UPML					93.0	4.6	0.2
PPML					90.1	8.8	0.5
UPLL					79.5	7.4	
PPLL					82.7	11.0	1.4

At the genus level, *Lactobacillus* (93.0%), *Gardnerella* (4.6%), and *Atopobium* (0.2%) were the three most abundant components of the vaginal microbiota at 32–34 weeks in the control group (UPML) and probiotic group before probiotics were administered (PPML). *Lactobacillus* (79.5%), *Gardnerella* (7.4%), and *Leptotrichia* (3.8%) were the three most abundant components of the prenatal vaginal microbiota at full term in the control group (UPLL). *Lactobacillus* (82.7%), *Gardnerella* (11.0%), and *Atopobium* (1.4%) were the three most common components of the vaginal microbiota at full term in the probiotic group after probiotics were administered (PPLL). The difference between the two groups was not significant (Table 3; Figure 7B).

In the probiotic group, *g_Blautia, s_Ruminococcus_sp_5_1_39BFAA, and g_Subdoligranulum* were significantly more abundant in the gut microbiota at full term than in that of the control group (*p* < 0.05) (Figure 7C).

### Variation in the placental microbiota in probiotic-treated pregnant patients

**Figure 8:**
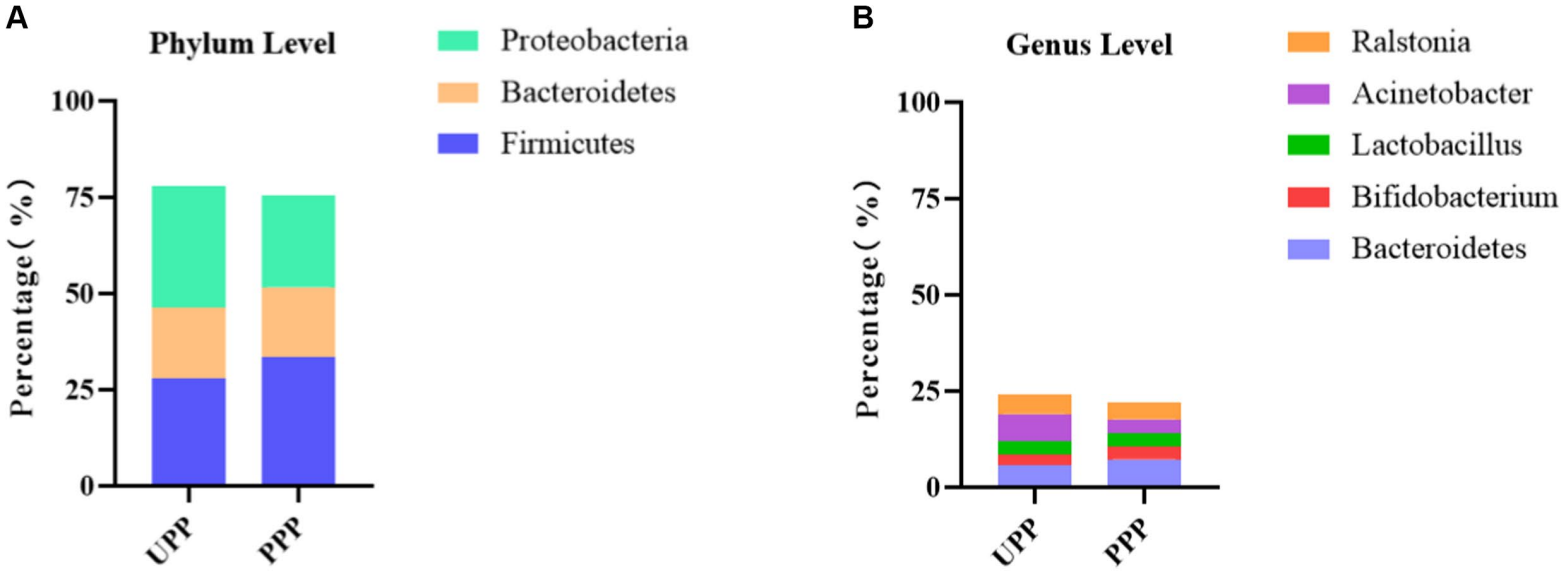
**(A)** Relative OTUs of the top three placenta microbiota at the phylum level. **(B)** Relative OTUs of the top five placenta microbiota at the genus level. UPP, placenta microbiota in the control group; PPP, placenta microbiota in the probiotic group.

At the phylum level, *Proteobacteria* (31.8%), *Firmicutes* (28.0%), and *Bacteroidetes* (18.3%) were the three most abundant components of the placental microbiota in the control group (placenta microbiota in the control group (UPP)). *Firmicutes* (33.4%), *Proteobacteria* (23.9%), and *Bacteroidetes* (18.3%) were the three most abundant components of the placental microbiota in the probiotic group [placenta microbiota in the probiotic group (PPP)]. The difference between the two groups was not significant (Table 4; Figure 8A).

**Table 4:** Relative OTUs of the top three placenta microbiota at the phylum level (%).

	*Firmicutes*	*Bacteroidetes*	*Proteobacteria*
UPP	28.0	18.3	31.8
PPP	33.4	18.3	23.9

At the genus level, *Acinetobacter* (7.1%), *Bacteroides* (5.8%), and *Ralstonia* (5.0%) were the three most abundant components of the placental microbiota in the control group (UPP). *Bacteroides* (7.3%), *Ralstonia* (4.5%), and *Actinobacteria* (3.7%) were the three most abundant components of the placental microbiota in the probiotic group (PPP) (Table 5; Figure 8B). The difference between the two groups was not significant (*p* < 0.05).

**Table 5:** Relative OTUs of the top five placenta microbiota at the genus level (%).

	*Bacteroidetes*	*Bifidobacterium*	*Lactobacillus*	*Acinetobacter*	*Ralstonia*
UPP	5.8	2.7	3.5	7.1	5.0
PPP	7.3	3.2	3.5	3.7	4.5

### The variation in the gut microbiota of newborns in the control group and probiotic group at different times

**Figure 9:**
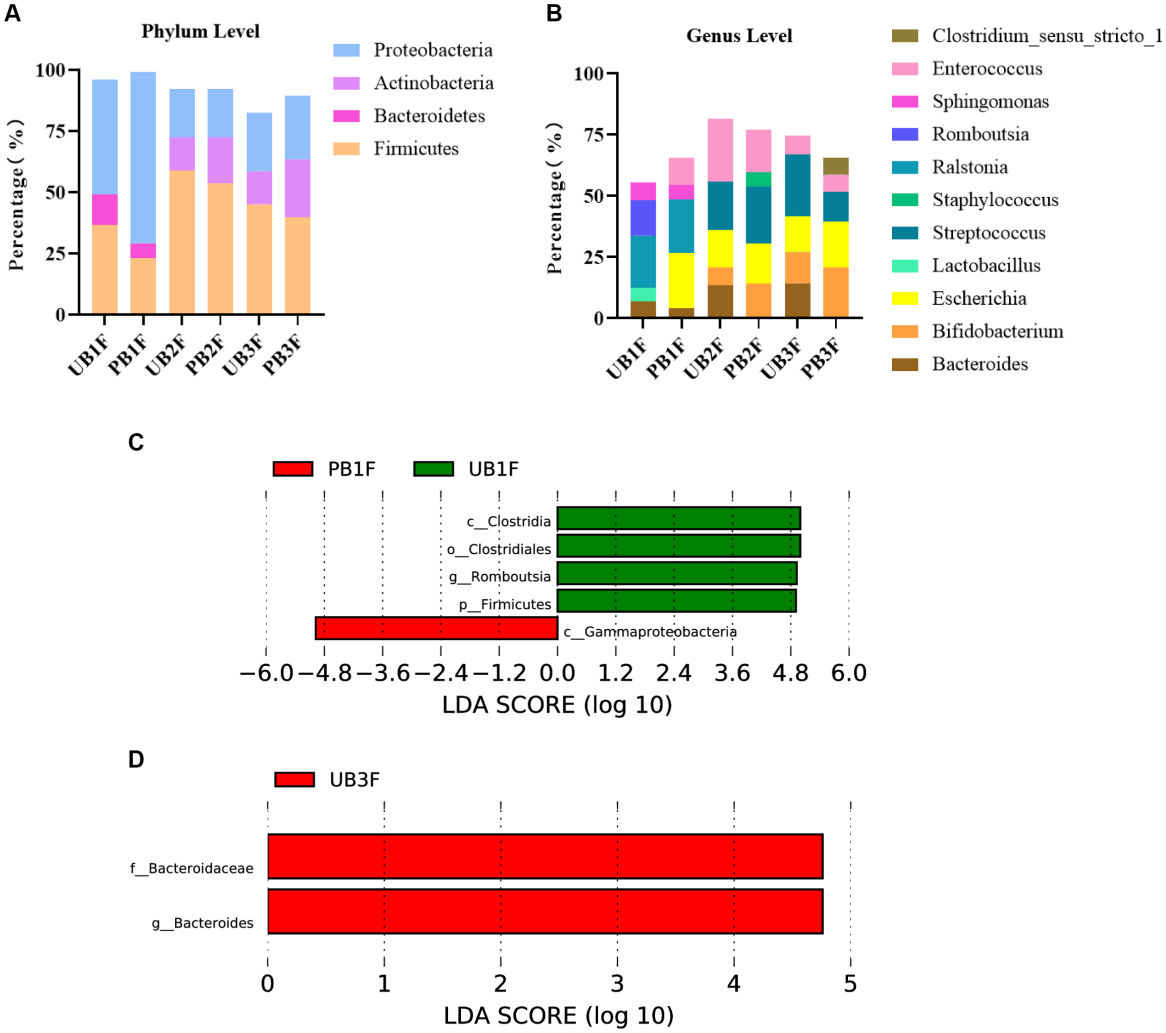
**(A)** Relative OTUs of the top three gut microbiota constituents of neonates at the phylum level. **(B)** Relative OTUs of the top five gut microbiota constituents of neonates at the genus level. **(C)** LDA analysis results for the meconium microbiota in the control group and the probiotic group. **(D)** LDA analysis results for the infant gut microbiota on the 14th day in the control group and the probiotic group. UB1F, meconium in control group; PB1F, meconium in probiotic group; UB2F, the gut microbiota of newborns on the 3rd day in control group; PB2F, the gut microbiota of newborns on the 3rd day in probiotic group; UB3F, the gut microbiota of newborns on the 14th day in control group; PB3F, the gut microbiota of newborns on the 14th day in probiotic group.

At the phylum level, *Proteobacteria*, *Firmicutes,* and *Bacteroidetes* were the three most abundant components of the meconium microbiota in both the control group [meconium microbiota in the control group (UB1F)] and the probiotic group [meconium microbiota in the probiotic group (PB1F)]. *Firmicutes*, *Proteobacteria,* and *Bacteroidetes* were the most abundant components in the gut microbiota on the 3rd and 14th day in both the control group [the gut microbiota of newborns on the 3rd day in the control group (UB2F) and the gut microbiota of newborns on the 14th day in the control group (UB3F)] and the probiotic group [the gut microbiota of newborns on the 3rd day in the probiotic group (PB2F) and the gut microbiota of newborns on the 14th day in the probiotic group (PB3F)] (Table 6; Figure 9A).

**Table 6:** Relative OTUs of the top three gut microbiota constituents of neonates at the phylum level (%).

	*Firmicutes*	*Bacteroidetes*	*Actinobacteria*	*Proteobacteria*
UB1F	36.6	12.4	/	47.1
PB1F	23.1	6.0	/	70.0
UB2F	58.9	/	13.5	19.8
PB2F	53.7	/	18.6	20.1
UB3F	45.1	/	13.6	23.9
PB3F	39.7	/	23.8	25.9

At the genus level, *Ralstonia* (21.3%), *Romboutsia* (14.5%), and *Sphingomonas* (7.2%) were the three most abundant components of the meconium microbiota in the control group (UB1F). *Escherichia* (22.4%), *Ralstonia* (22.0%), and *Enterococcus* (11.4%) were the most abundant components of the meconium microbiota in the probiotic group (PB1F) (Table 7; Figure 7B). *Enterococcus* (25.8%), *Streptococcus* (19.8%), and *Escherichia* (15.3%) were the three most common bacteria in the gut microbiota on the 3rd day in the control group (UB2F). *Streptococcus* (23.4%), *Enterococcus* (17.2%), and *Escherichia* (16.5%) were the three most abundant bacteria in the gut microbiota on the 3rd day in the probiotic group (PB2F) (Table 7; Figure 9B). *Streptococcus* (25.1%), *Escherichia* (14.6%), and *Bacteroides* (14.3%) were the three most abundant components of the gut microbiota on the 14th day in the control group (UB3F). Among the gut microbiota in the probiotic group (PB3F), *Bifidobacterium* (20.6%), *Escherichia* (18.8%), and *Streptococcus* (12.3%) were the three most common components on the 14th day (Table 7; Figure 9B).

**Table 7:** Relative OTUs of the top five gut microbiota constituents of neonates at the genus level (%).

	*Bacteroides*	*Bifidobacterium*	*Escherichia*	*Lactobacillus*	*Streptococcus*	*Staphylococcus*	*Ralstonia*	*Romboutsia*	*Sphingomonas*	*Enterococcus*	*Clostridium_sensu_stricto_1*
UB1F	6.8			5.6			21.3	14.5	7.2		
PB1F	4.1		22.4				22.0		5.8	11.4	
UB2F	13.3	7.4	15.3		19.8					25.8	
PB2F		14.0	16.5		23.4	5.8				17.2	
UB3F	14.3	12.8	14.6		25.1					7.7	
PB3F		20.6	18.8		12.3					7.0	6.7

LEfSe was used to estimate the difference between the gut microbiota of newborns in the control group and the probiotic group at different times. In the probiotic group, c*_Clostridia*, *o_Clostridiales*, *g_Romboutsia,* and *p_Firmicutes* were significantly less abundant in the meconium microbiota than in that of the control group (*p* < 0.05); *c_Gammaproteobacteria* were significantly less abundant in the meconium microbiota than in that of the control group (*p* < 0.05) (Figure 9C). The difference between the two groups on the 3rd day was not significant. *F_Bacteroidaceae* and *g_bacteroides* in the probiotic group were significantly less abundant in the gut microbiota than in the control group on the 14th day (*p* < 0.05) (Figure 9D).

## Discussion

The gut microbiota plays a key role in human health throughout life (1). However, we have limited information about their establishment in infants or their mechanism of action (17). Most studies indicate that microorganisms implanted in the newborn gut after birth are influenced by special factors, such as antibodies in the placenta, the umbilical cord (18), and maternal milk (19). Animal experiments have shown that the development of the nervous and immune systems of offspring is influenced by the maternal gut microbiota (20). This influence may be directly or indirectly caused by molecules or antibodies produced by the metabolism of the gut microbiota through the placenta or other pathways (21). In 2010, Maria reported that the gut microbiota of naturally born newborns was similar to that of mothers (22). However, a similar result was not found in cesarean-section pregnancies (22). Therefore, in our study, we analyzed 30 Chinese newborns at different stages. We found that meconium is not bio-clean. Meconium can harbor many microorganisms, such as *Proteobacteria*, *Actinobacteria,* and *Acidobacteria*. SourceTracker program has provided one of the most powerful and effective methods to perform microbial source tracking (15) and has been widely used (23–25). It uses Bayesian methods to evaluate all assignments of sink sequences to all source samples, including an unknown source, and creates a joint distribution of those assignments. Then, the distribution is sampled with a Gibbs sampler to estimate the likelihood that a sequence came from a particular source (26). In our study, we found that gut microbiota in meconium is composed mainly of bacteria from the placenta and an unknown source. We thought that there might be some “pathway” from the placenta to the neonatal gut to transfer bacteria. We also found that on the 3rd day and 14th day, neonatal gut microbiota tended to be composed mainly of bacteria from maternal gut microbiota and an unknown source. This trend is interesting. We thought that after birth, neonatal gut microbiota was still influenced by their maternal placenta at first. However, this influence decreased after disconnecting with the placenta. Interestingly, for some reason, the influence of the maternal gut increased. So, maternal gut microbiota is more vital to their offspring. This finding is controversial, as is our former recognition. Because the vagina and uterine are connected, we previously thought that vaginal microbiota may have more influence on neonatal gut microbiota. Furthermore, previous studies focused little on the co-occurrence of bacterial networks in maternal microbiota and neonatal microbiota. We found that the correlation of bacteria in both maternal microbiota and neonatal microbiota was complicated, especially in the placenta and meconium. Hence, influential factors on neonatal gut microbiota is worthing studied.

Recent studies have focused mainly on the relationship between adult gut microorganisms and disease based on 16S RNA sequencing (17). However, the factors influencing newborn gut microbiota in the early stages of life are still unclear (17). In 2015, 98 Swiss full-term newborns and maternal fecal samples were analyzed via metagenome sequencing by Fredrik (17). The authors found that delivery mode and breastfeeding play key roles in the establishment and evolution of newborn gut microbiota (17). They also found that α diversity increased and β diversity decreased before 3 years of age (17). However, an American study revealed that a child’s gut microbiota becomes stable before 1 year of age (27). This difference in the results might be influenced by race and region (27). In our study, we first analyzed the gut microbiota of Chinese newborns at different stages through 14 days after birth. After using Spearman’s rank correlation analysis, we found that the gut microbiota at different times was related to the maternal microbiota at the phylum and genus levels. However, the influence of the maternal microbiota on the newborn gut microbiota differed in terms of timing and duration. Interestingly, the gut microbiota of newborns on the 14th day had less relationship with maternal microbiota. We hypothesized that these bacteria from pregnant patients have indirect effects, not direct delivery, on their offspring, such as genetic inheritance or microenvironment influence, which becomes more apparent until the newborn gut microbiota becomes more stable (17). In 2015, Bäckhed found that the gut microbiota of 12-month-old children was more similar to that of their mothers than was that of newborns and 4-month-old children. This suggests that the maturation of the gut microbiota is a non-stochastic process that may result from the interactions that occur at different developmental periods between key microbial communities (28).

Many studies have shown that the maternal gut and vaginal microbiota at different stages can influence fetal or newborn gut microbiota (18) via the blood (19), placenta, or direct contact. This relationship could also influence the growth of the fetal nervous and immune systems (29–31). Several observational studies have shown that variations in a child’s gut microbiota can reduce the occurrence of allergic diseases (32). For instance, in 2021, several studies showed the influence of probiotics used by children on allergic diseases (12, 33). It is interesting that in our study, neonatal gut microbiota was more similar to placenta and maternal gut microbiota, especially gut microbiota of meconium. So, can we variate neonatal gut microbiota via maternal probiotic management? Probiotics are common medicines that influence gut microorganisms in newborns by increasing the diversity of the gut microbiota (32, 34). Previous studies showed that probiotics could work in approximately 2–4 weeks (35–37). *Bifidobacterium longum*, one of the most abundant microorganisms in the intestines of infants and adults, is usually used in probiotics (38). *Streptococcus thermophilus* (39) and *Lactobacillus delbrueckii bulgaricus* (40) are considered to be potential probiotics and have been used to promote human health (41, 42). However, few studies have investigated the variation in the gut microbiota of newborns via oral maternal intake of probiotics during pregnancy. In our study, we first administered oral probiotics to pregnant women at 32–34 weeks to investigate the influence of these probiotics on the gut and vaginal microbiota. We found that the abundance of *Bifidobacterium* increased in maternal gut microbiota and placenta microbiota. Abundance of *Lactobacillus* increased in maternal vaginal microbiota. However, there is no statistical difference for the small sample size. Furthermore, in the probiotic group, *g_Blautia, s_Ruminococcus_sp_5_1_39BFAA, and g_Subdoligranulum* were significantly more abundant in the gut microbiota at full term than in the control group. These findings are inconsistent with most of the previous studies. Several previous studies have shown that oral probiotics can alter the gut and vaginal microbiota to achieve therapeutic effects (43, 44). However, some previous studies have shown that probiotics cannot change the diversity or abundance of probiotic bacteria in the microbiota, but they can play a role (45–48). So, it is still controversial on the functional mechanism of probiotics. Even in previous studies with the conclusion that probiotic supplements could vary the abundance of probiotic bacteria, the abundance of other bacteria also could change (49). In our study, the abundance of some non-probiotic bacteria changed. Based on co-occurrence network analysis, we speculated that probiotics can change the microenvironment through their metabolites or receptor-mediated genes (50). However, determining the mechanism of this process will require further investigation.

In a previous study in 2016, Paul found that administering probiotics to pregnant and lactating rats changed the gut microbiota of their offspring and reduced the incidence of obesity in their offspring (51). In our study, the abundance of *Bifidobacterium* increased in neonatal gut microbiota in 3 days and 14 days in the probiotic group, but there is no statistical significance. However, some other bacteria in meconium and on the 14th day between the probiotic group and the control group had significant differences after probiotic management. Whether oral probiotics can change the gut microbiota of offspring is still controversial, too. Several studies have shown that probiotics can only affect Toll-like receptor expression in the placenta and in the newborn gut, not by affecting the constitution of the microbiota (52). In 2011, Boyle R investigated allergic disease in newborns in a randomized controlled trial of 250 pregnancies with high-risk factors. The authors found that probiotics administered to pregnant women after 36 weeks to full term could not prevent infantile eczema (53). Several studies have shown that probiotics administered from 36 weeks to 3 months after delivery can reduce the occurrence of infantile allergic dermatitis (54). These studies showed that probiotics taken after delivery may have a greater impact on newborns. In our study, based on co-occurrence network analysis, we speculated that probiotics also have an indirect effect on maternal and neonatal gut microbiota.

Our study has several limitations: first, the small sample size was the main limitation of the study. Second, we did not collect oral and mother milk microbiota. Third, further study is needed to investigate the relationship between maternal microbiota and their offspring’s microbiota after the 14th day.

## Conclusion

**Figure 10:**
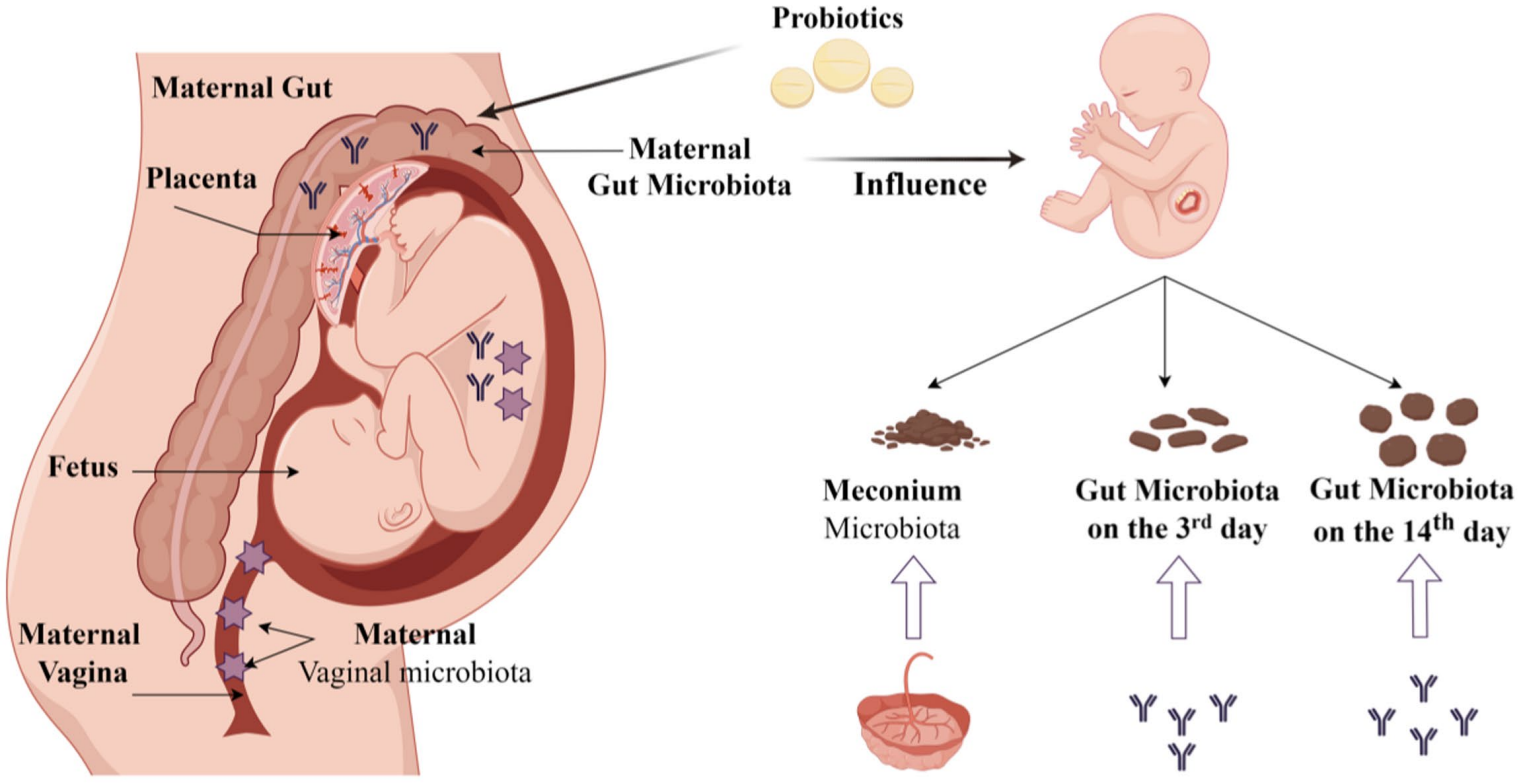
Origin of the neonatal gut microbiota and probiotic intervention (By Figdraw).

Maternal microbiota is vital to their offspring. In this study, we found that the microbiota in the neonatal gut at different times correlated with that in the maternal microbiota in the 3rd trimester and at full term. The placenta had more influence on meconium microbiota. The maternal gut had more influence on neonatal gut microbiota on the 3rd day and 14th day. Currently, the probiotic dosing we use during pregnancy does not alter the abundance of *Bifidobacterium, Lactobacillus, or Streptococcus* in the maternal gut, vaginal, and placental microbiota at the full term. However, we found that some other bacteria changed in the maternal gut and the neonatal gut in the probiotic group (Figure 10). We may need further study to investigate the mechanism of maternal-to-infant gut microbiota transmission.

## Data availability statement

The datasets presented in this study can be found in online repositories. The names of the repository/repositories and accession number(s) can be found at: https://www.ncbi.nlm.nih.gov/, PRJNA1053345.

## Ethics statement

The study project was authorized by the Institutional Review Board (IRB) for Human Subject Research at the First Affiliated Hospital of Jinan University (2019-011). The studies were conducted in accordance with the local legislation and institutional requirements. Written informed consent for participation in this study was provided by the participants’ legal guardians/next of kin.

## Author contributions

ZL: Funding acquisition, Investigation, Writing – original draft, Writing – review & editing. YZ: Methodology, Software, Writing – original draft, Writing – review & editing. XT: Data curation, Formal analysis, Writing – original draft. TK: Methodology, Resources, Writing – review & editing. QG: Methodology, Resources, Writing – review & editing. XX: Investigation, Methodology, Project administration, Writing – review & editing. CX: Investigation, Methodology, Project administration, Writing – review & editing.

## Footnotes

^1^ http://cutadapt.readthedocs.io/en/stable/

^2^ http://drive5.com/uchime/uchime_download.html

^3^ http://www.drive5.com/usearch/manual/uchime_algo.html

^4^ http://drive5.com/uparse/

^5^ https://www.arb-silva.de/

^6^ http://sourceforge.net/projects/rdp-classifier/

^7^ http://www.drive5.com/muscle/

^8^ http://www.mothur.org/wiki/Shannon

## Glossary

PMF: Gut microbiota at gestational 32-34 weeks women
PML: Vaginal microbiota at gestational 32-34 weeks women
PLF: Gut microbiota of gestational full-term women
PLL: Vaginal microbiota of gestational full-term women
PP: Placenta microbiota
B1F: Meconium microbiota
B2F: The gut microbiota of newborns on the 3rd day
B3F: The gut microbiota of newborns on the 14th day
UPMF: Gut microbiota at gestational 32-34 weeks women in control group
PPMF: Gut microbiota at gestational 32-34 weeks women in probiotic group
UPML: Vaginal microbiota at gestational 32-34 weeks in control group
PPML: Vaginal microbiota at gestational 32-34 weeks in probiotic group
UPLF: Gut microbiota of gestational full-term women in control group
PPLF: Gut microbiota of gestational full-term women in probiotic group
UPLL: Vaginal microbiota of gestational full-term women in control group
PPLL: Vaginal microbiota of gestational full-term women in probiotic group
UB1F: Meconium microbiota in control group
PB1F: Meconium microbiota in probiotic group
UB2F: The gut microbiota of newborns on the 3rd day in control group
PB2F: The gut microbiota of newborns on the 3rd day in probiotic group
UB3F: The gut microbiota of newborns on the 14th day in control group
PB3F: The gut microbiota of newborns on the 14th day in probiotic group
UPP: Placenta microbiota in control group
PPP: Placenta microbiota in probiotic group
OTU: Operational Taxonomic Units
